# Draft Genome Sequence of Clostridium perfringens *netB*-Positive Strain 2016TE7641_69, Isolated from the Intestine of a Diseased Turkey in Italy

**DOI:** 10.1128/MRA.00065-20

**Published:** 2020-05-14

**Authors:** Francesca Profeta, Elisabetta Di Giannatale, Massimiliano Orsini, Massimo Ancora, Camilla Smoglica, Cesare Cammà, Cristina E. Di Francesco

**Affiliations:** a Istituto Zooprofilattico dell’Abruzzo e del Molise G. Caporale, Teramo, Italy; b University of Teramo, Faculty of Veterinary Medicine, Teramo, Italy; University of Maryland School of Medicine

## Abstract

Here, we report the complete genome sequence of Clostridium perfringens 2016TE7641_69, isolated from the intestine of a turkey reared in a conventional poultry flock located in central Italy, where animals were showing enteric disorders suggesting subclinical necrotic enteritis.

## ANNOUNCEMENT

Clostridium perfringens is a Gram-positive, anaerobic, rod-shaped bacterium that is commonly detected in the environment and in the intestines of both mammals and birds (1). Based on its ability to produce several toxins (alpha to iota, C. perfringens enterotoxin [CPE], and NetB toxins), the classification of C. perfringens was recently extended to seven toxinotypes (2). C. perfringens type G is commonly recognized as the causative agent of avian necrotic enteritis, which is responsible for considerable economic losses in the poultry industry (3). However, many aspects of the disease, regarding both the pathogenesis and the virulence profile of the bacterium, remain unclear and need to be fully understood.

In this study, we sequenced the complete genome of the C. perfringens *netB*-positive field strain 2016TE7641_69. This strain was isolated from the intestine of a male turkey (38 days old), which had been raised in a conventional indoor flock located in Italy, after environmental sampling performed in 2016 (4).

A fragment of tissue was incubated anaerobically at 37°C for 24 h in 10 ml thioglycolate broth (5), and then 0.1 ml of broth was subcultured on 5% blood agar plates at 37°C for 24 h under anaerobic conditions. Presumptive C. perfringens colonies were confirmed by biochemical methods (bioMérieux, France) and selected for DNA extraction by means of a Maxwell instrument (Promega, Italy). Toxinotype G was identified by sequencing PCR amplicons of alpha-toxin- and NetB-encoding genes (4).

Library preparation was carried out using a Nextera XT library preparation kit (Illumina, Inc.) according to the manufacturer’s instructions. The libraries were loaded onto an Illumina 300-cycle NextSeq 500/550 midoutput reagent cartridge version 2 kit and sequenced on an Illumina NextSeq 500 platform, producing 150-bp paired-end reads.

Sequencing returned a total of 1,381,024 paired-end reads corresponding to a theoretical coverage of about 200× (expected genome size, 3.46 Mb). Default parameters were used for all software unless otherwise specified. Quality control, trimming, and preliminary assembly were performed in the Orione webserver (6) by using FastQC (http://www.bioinformatics.babraham.ac.uk/projects/fastqc), FASTQ positional and quality trimming (trimming the first 10 bases and imposing a nucleotide minimum quality score of 20 and a minimum length after trimming of 50 bases), and SPAdes version 3.11 (7), respectively. After the data were downloaded, contigs were refined using Pilon version 0.39 (8) and then ordered with ABACAS version 1.3 (9) using the genome of C. perfringens strain NCTC13170 as a guide (GenBank accession number LS483393). When possible, gaps in the pseudomolecule thus obtained were filled using GapFiller (10). The final assembly consisted of 15 contigs accounting for a total of 3,228,139 bp (*N*_50_, 448,963 bp; GC content, 27.65%). The annotation, performed by the NCBI staff using PGAP version 4.6 (11), returned 2,757 protein-coding genes, 8 rRNA gene operons, 84 tRNAs, 4 noncoding RNAs, and 54 pseudogenes.

Two large contigs that were not included in the assembly were assigned to plasmid sequences. They were checked for circularity by mapping reads on both edges using BWA version 0.7.17 with default parameters (12). The two plasmids, named pl2-2016TE7641_69 (GenBank accession number MN503253) and pl4-2016TE7641_69 (GenBank accession number MN503254), were finally assembled for 82,669 bp and 72,615 bp, respectively. They were annotated with Prokka version 1.12 (13), which returned 79 and 78 protein-coding genes for pl2-2016TE7641_69 and pl4-2016TE7641_69, respectively.

The antibiotic resistance genes *stp1* (multidrug resistance) (contig accession number RBXV01000015, position 560723.561442), *mprF* (multiple peptide resistance factor) (contig accession number RBXV01000014, nucleotide position 230924.233479; GenBank accession number MN503253, position 48628.48936; GenBank accession number MN503254, position 54484.56999), *rpoB* (rifampin resistance) (contig accession number RBXV01000015, position 1302371.1306075), and *bacA* (bacitracin resistance) (contig accession number RBXV01000015, position 785861.786295) and a TetR/AcrR-family transcriptional regulator (contig accession number RBXV01000001, positions 42404.42973 and 81514.82032; contig accession number RBXV01000004, position 112962.113540; contig accession number RBXV01000007, position 322160.322714) were identified using GeneMarkS (http://exon.gatech.edu/GeneMark/genemarks.cgi) and BLAST (https://blast.ncbi.nlm.nih.gov/Blast.cgi) tools. Sequence similarity searching revealed the virulence factors GroEL (contig accession number RBXV01000015, position 1174579.1176198), alpha-clostripain (*cloSI*) (contig accession number RBXV01000007, position 428639.430207), and NetB (GenBank accession number MN503253, position 30079.31047), enterotoxins (contig accession number RBXV01000007, position 143855.145516; contig accession number RBXV01000015, position 11975.14842), hemolysins (GenBank accession number MN503253, positions 41866.42048 and 42175.42519), and iota (*iap*) (contig accession number RBXV01000015, position 143676.145349) and beta2 (GenBank accession number MN503254, position 32200.32997) toxins.

### Data availability.

The C. perfringens strain 2016TE7641_69 draft genome was deposited in GenBank with the accession number RBXV00000000. The plasmid sequences were deposited in GenBank under the accession numbers MN503253 and MN503254. The raw reads were submitted to the SRA database under the accession number PRJNA490323.
